# The Use of Rabbit Bone in Surgery

**Published:** 1898-11-12

**Authors:** 


### THE USE OF RABBIT BONE IN SURGERY.

A case was shown by Mr. Watson Cheyne at the last meeting of the Clinical Society in which freshly-removed rabbit bone was used to replace a loss of bone which had been suffered by a patient some years previously. The patient was a youth who, eight years before, had lost large portions of his nasal bones in consequence of an accidental injury. In January of this year Mr. Watson Cheyne performed an operation for the relief of the deformity. He first raised a flap of skin from the right side of the nose, going down to and freely exposing the periosteum. He then removed the femur from the rabbit, split it into several pieces, and laid these on the exposed periosteum, replacing the flap so as to cover them in. A few days after the operation a little serum had to be let out, but the wound healed without suppuration, and the result was excellent.

